# Immunogenicity of *Trypanosoma cruzi* Multi-Epitope Recombinant Protein as an Antigen Candidate for Chagas Disease Vaccine in Humans

**DOI:** 10.3390/pathogens14040342

**Published:** 2025-04-03

**Authors:** Christian F. Teh-Poot, Andrea Alfaro-Chacón, Landy M. Pech-Pisté, Miguel E. Rosado-Vallado, Oluwatoyin Ajibola Asojo, Liliana E. Villanueva-Lizama, Eric Dumonteil, Julio Vladimir Cruz-Chan

**Affiliations:** 1 Laboratorio de Parasitología, Centro de Investigaciones Regionales “Dr. Hideyo Noguchi”, Universidad Autónoma de Yucatán, Mérida 97000, Mexico; 2 Department of Chemistry and Biochemistry, Hampton University, Hampton, VA 23668, USA; 3 Department of Tropical Medicine and Infectious Disease, Celia Scott Weatherhead School of Public Health and Tropical Medicine, Tulane University, New Orleans, LA 70112, USA

**Keywords:** Chagas disease, immunoinformatic, multi-epitope, HLA-A*02:01, Chagas patients

## Abstract

Chagas disease, caused by the protozoan *Trypanosoma cruzi* (*T. cruzi*), is the most significant neglected tropical disease affecting individuals in the Americas. Currently, available drugs, such as nifurtimox and benznidazole (BZN), are both toxic and ineffective in the chronic phase of the disease. A promising alternative is the development of a Chagas disease vaccine, although this effort is hampered by the complexity of the parasite and HLA polymorphisms. In addition, the activation of epitope-specific CD8^+^ T cells is critical to conferring a robust cell-mediated immune response and protection by producing IFN-γ and perforin. Thus, the antigen (s) for the development of a Chagas vaccine or immunotherapy must include CD8^+^ T cell epitopes. In this study, we aimed to develop a multi-epitope recombinant protein as a novel human vaccine for Chagas disease. Sixteen database programs were used to predict *de novo* 40 potential epitopes for the HLA-A*02:01 allele. Nine out of the 40 predicted epitopes were able to elicit IFN-γ production in Peripheral Blood Mononuclear Cells (PBMCs) from Chagas patients. Molecular docking revealed a good binding affinity among the epitopes with diverse HLA molecules. Therefore, a recombinant multi-epitope protein including these nine *T. cruzi* CD8^+^ epitopes was expressed and demonstrated to recall an antigen-specific immune response in *ex-vivo* assays using PBMCs from Chagas patients with the HLA-A*02 allele. These findings support the development of this multi-epitope protein as a promising candidate human vaccine against Chagas disease.

## 1. Introduction

Chagas disease, caused by the parasite *Trypanosoma cruzi*, is a neglected tropical disease endemic to the Americas. As per the World Health Organization, this disease affects 6–7 million people globally, with 70 million individuals at risk, primarily among those residing in poverty-stricken and endemic regions of South, Central, and North America [1]. In endemic regions, the parasites are predominantly transmitted through the feces of triatomine vectors during feeding, while in other parts of the world, most transmission can occur via blood transfusion or organ transplants [2]. During the acute phase, treatment for infected patients with benznidazole or nifurtimox is effective. However, these medications exhibit toxicity and reduced efficacy during the chronic phase of the disease [3]. Despite the efforts, there is no vaccine or immunotherapy for Chagas disease. The CD8^+^ T cells play a crucial role in the design of antigens for the development of vaccines, as they can recognize *T. cruzi* epitopes via Human Leucocyte Antigen (HLA) molecules, triggering the secretion of IFN-γ and perforin/granzyme. However, the diversity of the HLA molecules and the complexity of parasites have greatly hampered the identification of novel *T. cruzi* antigens [4]. *T. cruzi* proteins have been identified containing epitopes for the HLA-A*02:01 allele, including Kinetoplastid membrane protein (KMP-11) [5], Amastigote surface proteins (ASP-1 and ASP-2) [6], Trans-sialidase (TSA-1) [6], Calcium-binding protein (CaBP) [7], LYT-1 [7], Paraflagellar rod proteins (PFR-3 and PFR-2) [8], Heat shock protein (HSP70) [9], Flagellum-associated protein (FL-160) [10], Cruzipain [10], and Ribosomal P2 protein (TcP2b) [10].

Conventional methods to discover new antigens require a large amount of laboratory work, which is time-consuming and costly. Alternatively, immunoinformatic database analysis accompanied by molecular docking has been shown to be a robust strategy to identify CD8^+^ T cell epitopes and design multivalent vaccines against parasites and other pathogens such as viruses or bacteria [11,12,13,14,15]. The activation of antigen-presenting cells expressing HLA class I molecules presents a restricted set of *T. cruzi*-derived 8–11 amino acid peptides to CD8^+^ T cells. In this context, targeting these immunogenic epitopes could allow for the design of novel multi-epitope antigens to be used as an immunotherapeutic strategy to enhance the magnitude of the T cell response, potentially controlling *T. cruzi* infection and reducing the progression of Chagas disease cardiomyopathy.

Multi-epitope antigens have been designed using bioinformatics tools derived from the membrane or secreted proteins of *T. cruzi* [16,17,18]. Our initial attempt to develop and test a multi-epitope recombinant protein containing ten nonameric CD8^+^ epitopes using the Tc24-C4 protein as a scaffold in a proof-of-concept study. In mice, the multi-epitope recombinant protein induced an immune response mediated by antigen-specific CD8^+^ T cells producing IFN-γ and IL-4 cytokines, ameliorating the *T. cruzi* acute infection [19]. Furthermore, our group identified a set of 114 proteins as potential antigens, containing 150 predicted epitopes selected based on their binding score to H-2K^d^ and H-2D^d^ alleles using immunoinformatic analysis of the whole *T. cruzi* genome. The validated epitopes were found to have putative functions, such as metabolic enzymes or structural proteins of the cytoskeleton, and hypothetical proteins [20]. In addition, these proteins can be highly immunogenic and potential vaccine candidates to use in humans. About 24–33% of humans possess the HLA-A*02:01 allele, which is most prevalent in Latin American endemic areas for Chagas disease. Consequently, epitopes eliciting an immune response in HLA-A02:01 individuals are likely to be immunogenic in a significant portion of the afflicted human population [21,22]. Thus, to develop a multi-epitope vaccine candidate for humans, we identified HLA-A*02:01 epitopes of these 114 *T. cruzi* proteins identified in our previous work [20] and 14 additional proteins from literature. Next, we validated the CD8^+^ epitopes in PBMCs from chagasic patients, and those with IFN-γ production were included in the design of a novel multi-epitope recombinant protein. Finally, we assessed the multi-epitope recombinant protein to recall antigen-specific immune response in PBMCs from chagasic patients with the HLA-A*02:01 allele.

## 2. Materials and Methods

### 2.1. Trypanosoma cruzi Epitope Prediction from Databases

Sequences of the 114 *T. cruzi* proteins (version 3.3) were obtained from TriTrypDB (http://tritrypdb.org, accessed on 12 February 2019), which corresponds to the proteome of *T. cruzi* strain CL Brener Non-Esmeraldo. These proteins contained the 150 high-scoring epitopes with strong affinity for MHC in BA.

LB/c mice [20]. Additionally, we included 14 antigenic proteins of *T. cruzi* recognized by PBMC of patients HLA-A*02:01 with the potential to be vaccine candidates through literature search. All antigenic protein sequences were retrieved from their respective report.

In the first step, the 128 sequences were analyzed to predict epitopes (9–11 mer) for the HLA-A*02:01 allele using the NetMHC.3.2 [23] and RankPep [24] servers. Following this, the consensus was calculated based on the average score of the two servers for each peptide analyzed. Predicted peptides were selected to have average scores of ≥75% of maximum binding. In a second step, protein sequences containing peptides with ≥75% affinity were re-analyzed in 14 different servers to predict the affinity of the peptides to HLA-A*02:01, including SYFPEITHI [25], BIMAS [26], ProPred-I [27], ANPred [28], ComPred [28], PredEp [29], IEBD [30], CTLpred [31], MHCpred [32], Epijen [33], NetCTL [34], NetCTLpan [35], NetCTLcons [36], and HLArestrictor [37]. A second consensus combined the results from the different software (including RANKPEP and NetMHC 3.2 servers) to calculate an average rank of prediction for each epitope [38]. Peptides predicted by the highest number of programs and average rank ≤ 4.0 were prioritized for successive analysis. In a third step, peptide sequences showing >80% similarity to human or mouse proteins using pBLAST [39] were discarded from further analysis to avoid potential autoreactivity. This gave a set of 40 peptides, and they were taken forward.

### 2.2. Epitope Validation

The 40 peptides with the highest binding prediction score to HLA-A*02:01 were synthesized by Peptide 2.0 Inc. (Chantilly, VA, USA). The synthetic peptides (72–99% purity) were dissolved in water or DMSO at a concentration of 5 mg/mL and stored at −80 °C until use. The peptides were validated in PBMCs from chagasic patients and healthy individuals with HLA-A*02:01 upon stimulation with synthetic peptides. A total of 22 *T. cruzi* infected patients and 19 uninfected controls were HLA typed, resulting in our HLA-A*02 adult Chagas disease patients and three healthy donors that were enrolled. All participants reside in Yucatan, Mexico, and patients had never received treatment for the disease. The HLA typing and *T. cruzi* infection of all participants were reported in a previous study [40]. All healthy controls were negative to all serological tests. No clinical alterations were found in chagasic patients and classified in the asymptomatic chronic phase [40].

All participants were sampled collecting 24 mL of peripheral blood by venous puncture in tubes with heparin for PBMC isolation. The PBMCs were obtained by centrifugation using the density gradient separation technique with Ficoll Histopaque-1077 (GE Healthcare, Uppsala, Sweden) [40]. Briefly, the peripheral blood was diluted 1:1 with Dulbecco’s phosphate-buffered saline (DPBS) pH 7.4 (Invitrogen, MA, USA). Blood diluted was added to a Ficoll Histopaque at a 2:1 ratio and centrifuged at 400× *g* for 40 min. The PBMC layer was separated and washed twice with PBS pH 7.4 (Invitrogen). Finally, the PBMCs were suspended in RPMI medium (RPMI GlutaMAX-I medium-Gibco, Grand Island, NY, USA) supplemented with 10% fetal bovine serum (Gibco, New York, NY, USA) and 1% penicillin/streptomycin (Gibco, New York, NY, USA). The PBMCs were counted in a Neubauer chamber, and viability was determined with trypan blue staining (Sigma, Taufkirchen, Germany).

For in vitro stimulation, we used 2.5 × 10^5^ PBMC/well in a 96-well culture plate and stimulated with 5 µg/mL of each individual synthetic peptide for 24 h at 37 °C in 5% CO_2_. PBMCs stimulated with 20 µg/mL of Phytohemagglutinin (PHA) (Roche, Mannheim, Germany) or 2 µg/mL of CEF (Cytomegalie, Epstein Barr, and Influenza virus) (Mabtech, Nacka Strand, Sweden) were used as positive controls, and unstimulated PBMCs were used as negative controls (RPMI). The CEF pool of 23 peptides was used as a reliable control to detect levels of IFN-γ produced by CD8 memory T-cell responses in PBMCs from patients with HLA-A*02. After 24 h, supernatants were collected, and interferon-gamma (IFN-γ) levels were measured using a cytometric bead array (CBA) according to the manufacturer’s instructions (CBA Flex Set, BD Biosciences, San Diego, CA, USA). Samples were processed following the manufacturer’s instructions. Briefly, 50 μL of supernatant aliquots were incubated for 2 h with the capture bead, following a 2 h incubation with PE-detection reagent, then washed with wash buffer and centrifuged at 200× *g* for 5 min. Samples were acquired in a FACSVerse cytometer, and IFN-γ concentrations were determined using FCAP Array v3.0 software. A peptide was considered an epitope when values were above the limit of detection (0.8 pg/mL) and higher than the value produced by healthy donors.

### 2.3. Trypanosoma cruzi-Epitopes Conservation

The conservation of the epitopes among diverse strains and discrete typing units of *T. cruzi* was evaluated using sequences from the TriTrypDB database. The selected T cell epitope sequences were analyzed among *T. cruzi* strains Brazil A4, Dm28c (DTU I), Sylvio X10/1, G (DTU I), CL Brener Esmeraldo-like, Y C6 (DTU II), CL Brener Non-Esmeraldo-like (DTU III), TCC (DTU VI), and CL Brener (DTU VI) [41]. Epitopes showing 100% of sequence identity were considered conserved. Additionally, we identify the similarity of the nine residues among protein sequences of kinetoplastid species using pBLAST. Epitopes showing >77% (7 out of the 9 residues) sequence identity were reported.

### 2.4. Docking of T. cruzi Epitopes to HLA Class I Molecules

The promiscuous epitopes to common HLA alleles in the Mexican and Latin American populations were analyzed by molecular docking. For this, the alleles A*01:01 (4NQX), A*02:01 (5SWQ), A*02:06 (3OXR), A*03:01 (6O9B), A*11:01 (5WJL), A*24:02 (5WWJ), A*30:01 (6J1W), A*68:01 (6PBH), A*68:02 (4I48), B*07:02 (7LG0), B*08:01 (3 × 13), B*14:02 (3BVN), B*15:01 (6VB3), B*18:01 (4JQV), B*27:05 (6VQE), B*35:01 (4LNR), B*39:01 (4O2E), B*40:01 (6IEX), B*40:02 (6AT5), B*44:02 (3DX6), B*44:03 (3KPN), B*51:01 (1E28), B*52:01 (3W39), B*53:01 (1A1O), and B*57:01 (2RFX) were submitted in CABSdock with nine *T. cruzi*-epitopes to analyze the interactions by molecular docking [42]. Interactions among epitopes and HLA were modeled by the CABS-dock [43]. The output solutions of CABSDock were subjected to the FireDock server, and the global energy was determined [44]. The Protein Data Bank (PDB) (https://www.rcsb.org/, accessed on 21 March 2020) was used to retrieve the crystal structures of HLA molecules [45]. To establish an epitope interaction in the specific binding groove of HLA, we considered three parameters: (1) RSMD (root mean square deviation calculated on the peptide after superposition of HLA molecule) [43], (2) energy of binding affinity [44] and (3) number of epitope interactions with peptide binding pockets of HLA class I [46]. GraphPad Prism software was used to draw a heat map showing the epitope interaction of each docking with each HLA molecule. Colors represent interaction, high interaction with green (3 out of the 3 parameters), no interaction in red (0 out of the 3), and changes in coloring from red to green, representing an increase in interaction. HLA molecules with high interaction were used in the next analysis.

### 2.5. In Silico Design of the Multi-Epitope Recombinant Protein

The epitopes with the ability to induce IFN-γ production by PBMCs from patients with HLA-A*02 were used to design the multi-epitope protein. To construct the multi-epitope protein, epitopes were intercalated using the spacer sequence EEKK, increasing solubility and avoiding interaction among epitopes. His-tag was added to the C-terminus of the multi-epitope protein sequence for the purification process. Next, the multi-epitope sequence was analyzed in Biotech, Solpro (threshold < 0.5), and Protein-sol (threshold < 0.5) to predict the solubility in the *E. coli* expression system [47,48,49]. The TMHMM server was used to predict the presence of the transmembrane domains in the multi-epitope protein sequence because these domains in the protein could hinder the yield in the purification process [50]. Additionally, ProtParam was used to evaluate physicochemical properties, such as amino acid composition, theoretical isoelectric point (pI), and molecular weight [51]. The designed multi-epitope protein was subjected to the VaxiJen for antigenicity evaluation [52]. The allergenic potential of the multi-epitope protein was analyzed by AllerTOP.2 and AlgPred [53,54]. In addition, the 3D structure of the multi-epitope protein was modeled using the Raptor-X server [55]. The population coverage (PC) is defined as the fraction of individuals in a population that responds to the epitope. The IEDB population analysis tool (http://tools.iedb.org/population/ accessed on 19 Novemeber 2021) was used to estimate the PC of HLA-A*02:01 epitopes in Mexico and South American populations [56]. The PC was assessed based on the HLA molecules with high interaction for each epitope, which were predicted by molecular docking analysis.

### 2.6. Expression of the Multi-Epitope Protein

The multiepitope protein was expressed and purified by Genescript Inc. (Piscataway, NJ, USA). Briefly, the gene-encoding the multi-epitope protein was synthesized and cloned into vector pET14a using NdeI and EcoRI restriction sites. Next, *E. coli* Rosetta 2 (DE3) and BL21 Star (DE3) strains were transformed with the plasmid pET-41a (+)-multi-epitope. The transformed bacteria were grown in LB broth containing kanamycin, and expression was induced by the addition of 0.5 mM Isopropyl β-D-1-thiogalactopyranoside (IPTG) for 16 h at 15 °C or 3 h at 37 °C. Collected cells were lysed (50 mM Tris-HCl, 0.5 M NaCl, 8 M urea, pH 8.0) and homogenized by sonication. The whole cell lysate was collected and passed through a Ni-NTA affinity column for purification. The multi-epitope protein was eluted under denaturing conditions (50 mM Tris-HCl, 0.15 M NaCl, 8 M urea, 50 mM imidazole, pH 8.0). The integrity and size of the multi-epitope protein were analyzed by 4–20% Bis-Tris SDS-PAGE (sodium dodecyl sulfate polyacrylamide gel electrophoresis) (Genscript, Piscataway, NJ, USA), and western blot analysis was performed using an anti-His-tag antibody. The multi-epitope recombinant purified protein was dialyzed in PBS overnight at 4 °C following decreasing concentrations of urea. The multi-epitope protein concentration was determined by Bradford’s assay and stored until used.

### 2.7. Multi-Epitope Recombinant Protein Validation

To confirm the immunogenicity of the multi-epitope, we used a recall response assay. For this, PBMCs from chagasic patients with the HLA-A*02 allele (n = 3) and healthy donors (n = 3) were isolated, as described above. The PBMCs were seeded onto a 96 well plate at 2.5 × 10^5^ cells/well and stimulated with 100 µg/mL or 50 µg/mL of the multi-epitope protein or pool of nine epitopes for 24 h at 37 °C in a 5% CO_2_ incubator. We used Concanavalin A (ConA) (5 µg/mL) (Sigma, Taufkirchen, Germany) and soluble *T. cruzi* antigen (20 µg/mL) as positive controls and unstimulated PBMC as a negative control. Culture supernatants were collected, and IFN-γ levels were measured using a CBA (cytometric bead array) according to the manufacturer.

### 2.8. Data Analysis

The results were presented using individual point graphs with mean ± SEM. To compare the means of IFN-γ levels among Chagasic patients and healthy donors, we used the Mann–Whitney U test. Graphs were constructed and statistical analyses were performed using GraphPad Prism 9 software. Differences were considered significant for *p* values < 0.05.

### 2.9. Ethical Considerations

The protocols were approved by the Ethical Committees at the Regional Research Center “Dr. Hideyo Noguchi” of the Autonomous University of Yucatan (Num.CEI-09-2019). A signed informed consent (Supplementary Figure S1) was obtained from all participants prior to the study. To validate the immunogenicity of epitopes, blood samples were collected in the period November 2019–February 2020. To validate the immunogenicity of the multi-epitope protein, blood samples were collected in February 2022.

## 3. Results

### 3.1. Identification of T. cruzi Epitopes to HLA-A*02:01

To identify *de novo* CD8^+^ T cell epitopes, the 128 selected protein sequences from previous studies were re-analyzed in RankPep and NetMHC to predict epitopes for the HLA-A*02:01 allele. RANKPEP predicted 297 out of the 99,534 peptides with a binding score > 70% for HLA-A*02:01 and NetMHC 1316 out of the 367,548 peptides. In total, 175 out of 1613 peptides were retrieved based on the first screening with the RankPep and NetMHC programs (Supplementary Figure S1A). In the second step of the analysis, the 64 protein sequences containing the 175 peptides were further analyzed with an additional 14 databases to predict epitopes for the HLA-A*02:01 allele. Here, we selected 46 epitopes predicted at least by 12 out of 16 programs (Supplementary Figure S1B), with average ranks of prediction among 1.0–4.7. In the third step, six out of the 40 peptide sequences were discarded because they showed more than 80% similarity to human or mouse sequences in the pBLAST database, indicating a potential risk of generating autoimmune responses. Finally, forty predicted epitopes were synthesized for in vitro validation using PBMCs from chagasic patients with the HLA-A*02 allele (Supplementary Table S1). Interestingly, 21 out of 40 peptide sequences were in proteins with putative functions such as metabolic enzymes or structural proteins, and 13 out of 40 were hypothetical proteins.

### 3.2. Epitopes Induced IFN-γ in PBMC from Chagasic Patients

**Figure 1:**
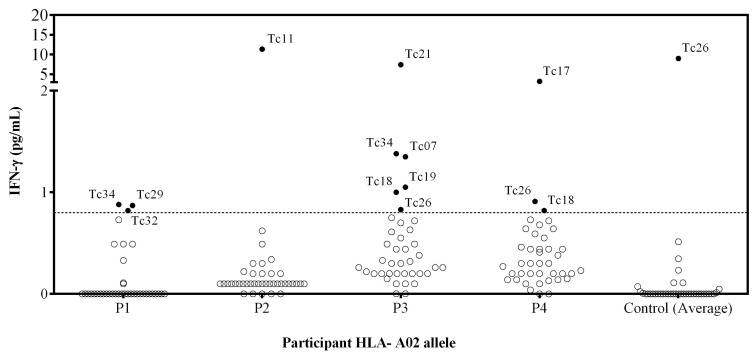
Assessment of the IFN-γ release by PBMC stimulated with individual peptides from patients with HLA-A*02 allele. PBMC from 4 *T. cruzi*-infected individuals and 3 *T. cruzi* seronegative subjects were stimulated with individual synthetic peptides, and cell supernatants were analyzed for IFN-γ release using a cytometric bead array. Empty circles represent the individual values for each peptide stimulation. Filled circles correspond to peptides inducing IFN-γ production. The control represents the mean (3 healthy donors) IFN-γ of the cells stimulated with each peptide. The dotted line corresponds to the limit of detection (0.8 pg/mL IFN-γ).

To validate the immunogenicity of the 40 predicted epitopes, IFN-γ production was assessed in vitro by stimulating PBMCs with the individual synthetic peptides. The IFN-γ production from PBMCs stimulated with PHA as a positive control of chagasic patients was 414 pg/mL, and for healthy donors, it was 435 pg/mL. Unstimulated PBMC showed no detectable IFN-γ production for any individual (Figure 1). Furthermore, stimulation with CEF peptides reveals detectable levels of IFN-γ produced by T cells (134 pg/mL), indicating the presence of CD8^+^ memory T cell responses in PBMCs from patients with HLA-A*02.

Ten out of 40 synthetic peptides (Tc07, Tc11, Tc17, Tc18, Tc19, Tc21, Tc26, Tc29, Tc32, and Tc34) induced a peptide-specific production of IFN-γ, and Tc26 was discarded because healthy donors presented a higher IFN-γ production than chagasic patients (Figure 1). The Tc11, Tc17, and Tc21 epitopes induced a higher level of IFN-γ production, while none of the four patients recognized the Tc35, Tc36, Tc37, Tc38, Tc39, and Tc40 epitopes. Interestingly, the Tc17 epitope was found in three different proteins involved in parasite differentiation and TcCLB.509713.10 proteins associated with differentiation contained both Tc17 and Tc29 epitopes.

The nine identified epitopes were demonstrated to be immunogenic in PBMCs from patients with the HLA-A*02:01 allele. Ten *T. cruzi* proteins containing these nine epitopes were two hypothetical proteins, and eight had a putative function. The proteins containing the Tc17, Tc21, Tc29, and Tc34 epitopes were also found to have epitopes for H-2D^d^ and H-2K^d^ validated in mice and showed an association with infection control in BALB/c mice [20].

### 3.3. Epitopes Conserved in Multiples DTUs of T. cruzi

The HLA-A*02:01 epitopes were analyzed using pBLAST to identify similarities among the *T. cruzi* DTUs and other kinetoplastid parasite species. Seven out of the nine epitopes (Tc07, Tc11, Tc19, Tc21, Tc29, Tc32, and Tc34) were conserved with 100% identity across *T. cruzi* DTUs TcI, TcII, TcIII, and TcVI. Conversely, the epitope Tc18 was found conserved exclusively in DTU TcIII and CLB Non-Es (Table 1). Moreover, the Tc07, Tc11, Tc21, Tc32, and Tc34 epitopes presented some level of similarity among other Kinetoplastid parasite species, including *Trypanosoma brucei*, *Trypanosoma vivax,* and *Leishmania* species (Table 1). Considering that these epitopes were conserved within strains of *T. cruzi* and other kinetoplastid parasites, they may induce protection against the wide diversity of *T. cruzi* strains and could be used for a pan-vaccine against kinetoplastid parasites.

### 3.4. Promiscuous Epitopes and Population Coverage

**Figure 2:**
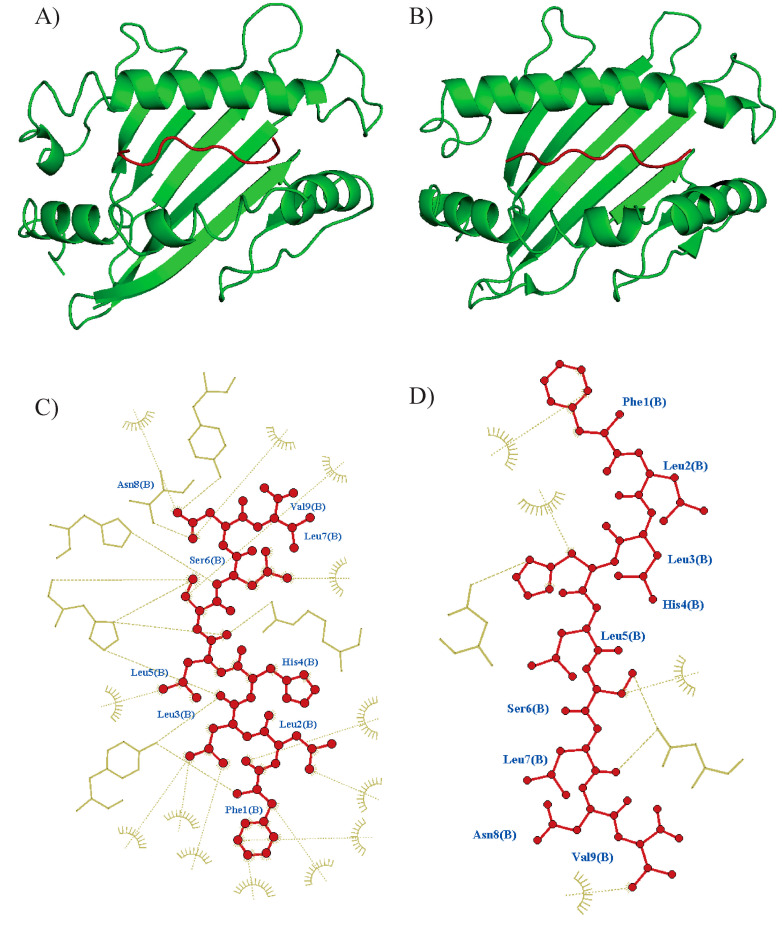
Overview of molecular docking among the epitope and HLA using CABSDock. PyMol was used to produce figures of docked complexes. (**A**) Tc07 epitope docking to HLA-A*02:01 predicted model (RSMD: 2.68, Binding affinity: −88 KJ mol^−1^) and (**B**) docking to A*52:01 (RSMD: 0.42, Binding affinity: −128 k mol^−1^). Epitope is shown in red color, and the HLA molecule is shown in green color. Interactions were visualized using Ligplus v.2.2 software, Tc07 epitope interaction to the respective binding site at (**C**) A*02:01 and (**D**) A*52:01. Interactions are shown with brown dashes.

**Figure 3:**
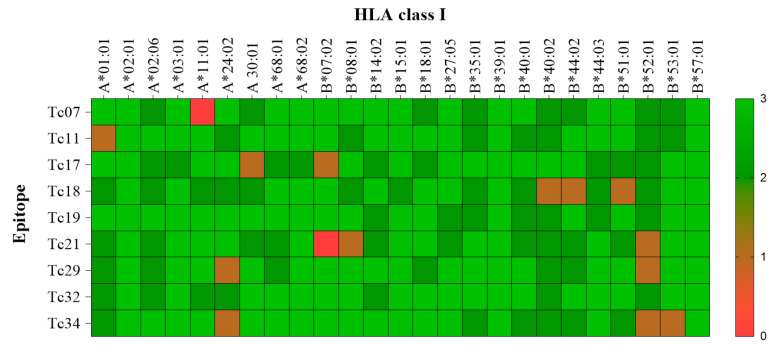
Heat map of the molecular docking interaction among epitopes with their respective HLA Alleles. The transversal axis of the heat map represents all the HLA I class available, and its sequence is determined by crystallography. The longitudinal axis represents the validated epitopes by IFNγ production in patients with A02:01. Colors represent the interaction based on the three parameters (RSMD, energy binding, and interaction force). Green represents high interaction (3 out of 3 parameters), and red represents no interaction (0 out of 3).

The promiscuity of epitopes was investigated using a molecular docking analysis (CABSdock server). The cut-off values for each parameter (RSMD ≤ 3.0, binding affinity ≤ −64.5 k mol^−1^, and number of interactions ≥ 22) were determined by the interaction of the nine epitopes with HLA-A*02:01 (Table 2). A representative docked interaction of the Tc07 in HLA-A*02:01 (high interaction) or B*57:01 (no interaction) is represented in Figure 2. Nine of the ten epitopes demonstrated promiscuous interactions with 11 out of 25 HLA class I molecules. Notably, Tc19 and Tc32 epitopes demonstrated the highest promiscuity, with interactions with 18 out of 25 HLA class I molecules. The HLA-B*39:01 and B*57:01 were predicted to have interactions with all epitopes (Figure 3). In addition, the epitope-HLA interactions were used to estimate the immunogenicity of the epitope in terms of population coverage based on the IEDB database. The Tc19 (93.5%) epitope had the highest predicted coverage for populations studied, Tc07 (83.5), Tc11 (85.4), Tc17 (81.7), and Tc18 (66.4). Tc19 (93.5), Tc21 (80.0), Tc29 (75.1), Tc32 (79.3), and Tc34 (83.9). The nine combined epitopes obtained a coverage of 97% and 77–99% of Mexican and Latin American populations, respectively.

### 3.5. Protein Expression of Multi-Epitope in E. coli

**Figure 4:**
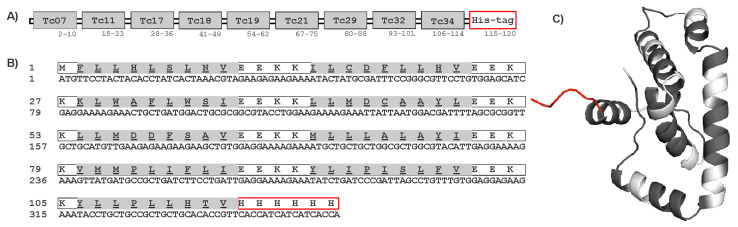
Construct of multi-epitope protein. (**A**) Schematic diagram of the final multi-epitope protein construct. (**B**) Amino acid and nucleotide sequences of ME protein. The amino acid sequence is shown in single-letter code, and HLA-A*02:01 epitopes are highlighted in gray. (**C**) The 3-dimensional structure of the multi-epitope protein with HLA-A*02:01 epitopes (gray), spacer EEKK (white), and His-tag added to the C-terminal (red).

The protein sequence and tertiary structure of the multi-epitope construct are shown in Figure 4. The multi-epitope protein was predicted to have solubility in the *E. coli* expression system using the Biotech, ProteinSol, and SOLpro servers. Transmembrane domains were also not found using the EEKK linker in the protein sequence by the THMM server. Other programs such as Vaxijen, AllerTop, and AlergPred predicted the multi-epitope protein sequence to be antigenic (score 0.56) and non-allergenic. Physicochemical properties predicted by the ProtParam server indicated that the multi-epitope contained 120 amino acids, with a molecular weight (MW) of 14.5 kDa and a 6.3 isoelectric point (pI). The multi-epitope protein was expressed in *E. coli* Rosetta by Genescript Inc. (Supplementary Figure S2A–C). The purified multi-epitope protein was analyzed by SDS-PAGE and Western blot as shown in Supplementary Figure S2B,C. The recombinant multi-epitope protein showed a band of approximately 14 kDa, and the yield was about 1.3 mg/L of culture medium.

### 3.6. Immunogenicity of the Multi-Epitope Protein in Chagasic Patients

**Figure 5:**
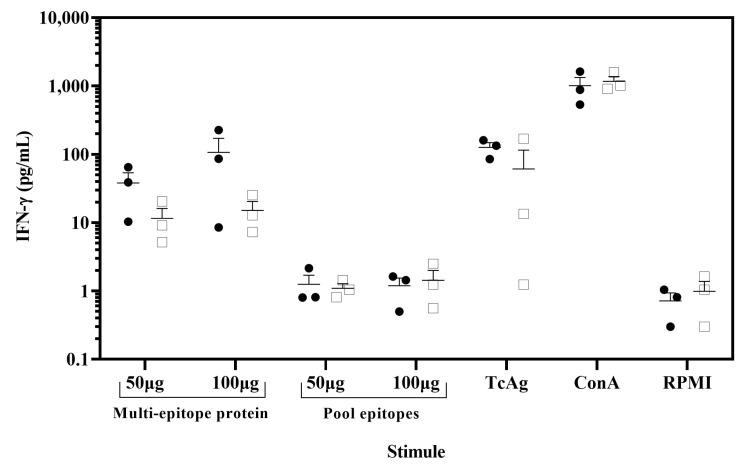
IFN-γ was released from *ex vivo* assay by stimulation of PBMCs with the multi-epitope recombinant protein. PBMC from chagasic patients (●, n = 3) and *T. cruzi* seronegative donors (□, n = 3) were incubated with 100 µg/mL or 50 µg/mL of the multi-epitope recombinant protein and pool of nine synthetic peptides. As positive controls, PBMC were stimulated with concanavalin A (ConA) (5 µg/mL) and soluble *T. cruzi* antigen (20 µg/mL) and unstimulated PBMC (RPMI) as negative controls. Cell supernatants were analyzed for IFN-γ release using a cytometric bead array. Data are presented as mean ± SEM for the concentration in pg/mL of IFN-γ, and horizontal lines indicate the mean.

To demonstrate the potential of the multi-epitope protein as a novel vaccine candidate, we evaluated its ability to induce a recall response in chagasic patients. The PBMCs from chagasic patients with HLA-A*02 were stimulated in vitro with the multi-epitope protein, and IFN-γ production was assessed by the CBA assay. The PBMCs stimulated with ConA as a positive control resulted in IFN-γ secretion reaching in patients and healthy donors 1012 and 1169 pg/mL, respectively. Importantly, PBMCs from chagasic patients exhibited a tendency to increase levels of IFN-γ when stimulated in vitro with the multi-epitope protein compared to the pool of nine synthetic peptides (Figure 5). In addition, PBMCs stimulated with the multi-epitope protein showed similar IFN-γ levels compared to soluble *T. cruzi* antigen (126 pg/mL). In sum, our data confirmed that the multi-epitope recombinant protein containing nine nonameric *T. cruzi* CD8^+^ epitopes for HLA A*02:01 was able to recall IFN-γ response in PBMC from chagasic patients and confirmed its potential as a novel vaccine candidate.

## 4. Discussion

The development of a Chagas disease vaccine is considered a promising alternative to prevent cardiomyopathy and control *T. cruzi* infection [57]. A multi-valent vaccine is preferable over a single antigen, as the latter is limited by the challenge of selecting a candidate antigen from among the nearly 12,000 proteins of *T. cruzi* [6,8,9,10,58,59,60]. To date, several multi-epitope proteins have been proposed as vaccine candidates against *T. cruzi* infection [16,17,18,19,61,62,63,64]. However, none of the candidates have been expressed and tested in *ex vivo* assays with *T. cruzi*-infected individuals, possibly hampered by the high polymorphism of HLA molecules [4]. Therefore, we focused the epitope prediction for the HLA-A*02:01 allele to rationally design a multi-epitope vaccine that can elicit a robust immune response via CD8^+^ T cells and IFN-γ production. The IFN-γ cytokine plays a crucial role in promoting the elimination of *T. cruzi*, as demonstrated in numerous studies [7,20,65,66,67,68]. Thus, robust immunoinformatic algorithms such as RankPep, NetMHC, and others 14 programs were employed to identify HLA-A*02:01 binding epitopes from 128 proteins that previously have been demonstrated to contain immunogenic murine H-2K^d^ and H-2D^d^ epitopes and resulted from the analysis of the whole *T. cruzi* genome [20].

Here, we identified and validated nine out of the 40 (22%) *T. cruzi* epitopes that induced IFN-γ production in PBMCs of chagasic patients. Previous studies have shown that PBMCs from asymptomatic patients, when stimulated with *T. cruzi* antigens, were able to elicit IFN-γ production. This finding suggests that these epitopes might be essential in triggering a protective immune response to prevent clinical progression. Therefore, these nine epitopes could be crucial in the development of therapeutic vaccines aimed at preventing or mitigating chronic symptomatic infections. The *T. cruzi* surface-proteins, including mucins, trans-sialidase (TS), and mucin-associated surface proteins (MASPs), are a key focus of research for the development of a therapeutic vaccine, as they represent a highly probable source of peptides for immune activation [69,70,71]. In our study, proteins containing these nine epitopes were recognizable with putative functions, including protein kinase, adenosine monophosphate deaminase, alpha/beta hydrolase, phospholipid- transporting ATPase, and proteins associated with parasite differentiation, parasite survival, and immune system evasion [20]. These findings confirm that other proteins may also serve as effective antigens for the development of a therapeutic vaccine. Further studies should confirm the expression of the two hypothetical proteins (TcCLB.508427.10 and TcCLB.504131.140) and explore their function in *T. cruzi* biology and immune activation. On the other hand, Tc35, Tc36, Tc37, Tc38, Tc39, and Tc40 epitopes induced undetectable levels of IFN-γ upon PBMCs re-stimulation and belonged to proteins previously identified as immunogenic in chronic chagasic patients [6,7,10]. However, several factors may contribute to the greater variability of recognition in subjects to the epitopes, including *T. cruzi* strain variability, the influence of exposure to other infections, or the exhaustion of T-cell response as a consequence of chronic antigen persistence [72,73,74].

Promiscuity of epitopes was investigated by their ability to bind to other HLA molecules in endemic populations with Chagas disease. We found that the nine epitopes showed high interaction with diverse HLA molecules, mainly A*03:01, A*68:02, B*39:01, and B*57:01, which are frequent in Mexico and Latin American populations [75]. Moreover, Tc17 and Tc29 epitopes were predicted to bind into 17 out of the 23 HLA alleles included in this study. On the other hand, HLA-B*39:01 has been associated with susceptibility and development of the symptomatic form of Chagas disease. Thereby, it is noteworthy that all nine epitopes were promiscuous with this allele and could potentially prevent clinical complications such as cardiomyopathy in these populations [76]. Further, the nine promiscuous epitopes may provide a wide coverage for the Mexican population (98%) or endemic areas of America Latin (77–99%) based on the IEDB database.

The variability of *T. cruzi* also represents a hurdle to developing a vaccine against Chagas disease. *T. cruzi* is subdivided into seven distinct discrete typing units (DTUs) with a high variability and diversity in the antigenic levels among the different strains [73,77]. Moreover, the diversity of the parasite significantly influences various aspects of its behavior and interaction with hosts. This variability can affect transmission rates, disease severity, and treatment efficacy [78]. Strikingly, the nine epitopes were conserved among the different DTUs of *T. cruzi* (TcI, TcII, TcIII, and TcVI) and other kinetoplastid parasite species. One limitation of this study is the absence of conserved epitopes among DTUs TcIV and TcV. This finding suggests that our study may not cover the full antigenic diversity present in *T. cruzi* strains, potentially impacting vaccine effectiveness, particularly in regions where TcIV and TcV are prevalent [79,80]. In fact, Tc17, Tc21, Tc29, and Tc34 epitopes were conserved in multiple DTUs (TcI, TcII, TcIII, and TcVI), and they also are in proteins associated with *T. cruzi* infection control in BALB/c mice [20]. Moreover, given that these epitopes are conserved among strains of *T. cruzi* and other kinetoplastid parasites, they could potentially provide protection against the wide diversity of *T. cruzi* strains and serve as a pan-vaccine for kinetoplastid parasites.

The multi-epitope design involved the selection of adequate spacers as GPGPG, AYYY, and EEKK. The spacers GPGPG and AYYY were employed in the construction of this multi-epitope protein. However, their utilization was deemed unsuitable due to the tendency to form transmembrane regions when incorporated as linkers. In contrast, the EEKK linker was determined to be more suitable for avoiding the formation of transmembrane regions and enhancing the solubility of the multi-epitope recombinant protein. The amino acid sequence KK within the spacer was specifically incorporated as it serves as the substrate for cathepsin B, a crucial protease involved in antigen processing [81].

The multi-epitope protein was determined to be non-allergenic and antigenic. To confirm this, we validated the immunogenicity of the multi-epitope protein by *ex vivo* recalling assay with PBMC from patients with HLA-A*02. Stimulation with both 50 and 100 µg of the multi-epitope recombinant protein elicited an antigen-specific immune response, as evidenced by increased levels of IFN-γ secretion from cells of *T. cruzi* infected individuals compared to the healthy donors. Our data suggested that protein containing the epitopes was appropriately processed by APCs to stimulate epitope-specific CD8^+^ T cells [82]. In addition, the multi-epitope protein induced higher levels of IFN-γ than the mixture of epitopes, which suggests that the use of epitopes as a multi-epitope protein improves the magnitude and quality of the immune response compared to individual epitopes, which would allow enhanced control of the infection. Conversely, the pool of individual epitopes used to stimulate the PBMCs was similar in Chagas patients and healthy donors. This could be attributed to the distinct hydrophobicity of individual peptides, which may mask the immunogenicity of the epitopes. In addition, the multi-epitope protein was capable of inducing IFN-γ, similar to other recombinant protein antigens (such as Tc24 or TSA-1) that have been tested in the same chronic patients with the HLA-A*02 allele, indicating the capacity of our multi-epitope protein to be a promising vaccine candidate. Due to the complexity of the immune system, further evaluation of IFN-γ production by CD8^+^ T cells would be crucial to confirm the potential of the multi-epitope protein as a vaccine candidate. Thus, our data represent an important step before future trials in non-human primates and human clinical trials.

In conclusion, these findings support the multi-epitope protein as a promising candidate vaccine against Chagas disease. While data suggest that the multi-epitope protein activates and recalls an antigen-specific immune response in patients with the HLA-A*02 allele, further validation in additional subjects is warranted, as well as the evaluation of the therapeutic efficacy using humanized murine models with appropriate alleles. Nonetheless, this study demonstrates that the combination of the immunoinformatic tools and the in vitro assays can be successfully used to design a multi-epitope protein as a first step towards future trials to develop a vaccine to control or prevent Chagas disease in humans.

## Figures and Tables

**Table 1:** Conservation of the novel HLA-A*02:01 epitopes among *T. cruzi* DTUs and kinetoplastids spp.

		Conservation		
ID	Epitope ^a^	Strain	DTU Tc ^b^	Kinetoplastids ^c^
Tc07	FLLHLSLNV	BrazilA4, Dm28c, SylvioX10, YC6, CLB Non-Es, TCC	I, II, III, VI	*T. brucei* (77), *L. mexicana* (78), *L. major* (78), *L. braziliensis* (88), *L. infantum* (78), *L. donovani* (78), *L. panamensis* (88)
Tc11	ILCDFLLHV	BrazilA4, Dm28c, SylvioX10, G, YC6, CLB Es, CLB Non-Es, TCC	I, II, III, VI	*T. brucei* (78), *T. vivax* (78)
Tc17	KLWAFLWSI	BrazilA4, Dm28c, SylvioX10, G, CLB Non-Es, TCC	I, III, VI	---
Tc18	LLMDCAAYL	CLB Non-Es	III	---
Tc19	LLMDDFSAV	BrazilA4, Dm28c, SylvioX10, G, YC6, CLB Non-Es, TCC	I, II, III, VI	---
Tc21	MLLLALAYI	BrazilA4, Dm28c, SylvioX10, YC6, CLB Es, CLB Non-Es, TCC, CLB	I, II, III, VI	*T. brucei* (78), *T. vivax* (78), *L. mexicana* (78), *L. major* (78), *L. braziliensis* (78), *L. infantum* (78), *L. donovani* (78), *L. panamensis* (78)
Tc29	VMMPLIFLI	BrazilA4, Dm28c, SylvioX10, YC6, CLB Non-Es, TCC	I, II, III, VI	---
Tc32	YLIPISLFV	BrazilA4, Dm28c, SylvioX10, YC6, CLB Es, CLB Non-Es, TCC	I, II, III, VI	*T. brucei* (88), *T. vivax* (88), *L. mexicana* (78), *L. major* (78), *L. braziliensis* (78), *L. infantum* (78), *L. donovani* (78), *L. panamensis*
Tc34	YLLPLLHTV	BrazilA4, Dm28c, SylvioX10, G, YC6, CLB Es, CLB Non-Es, TCC	I, II, III, VI	*T. brucei* (88), *L. mexicana* (78), *L. braziliensis* (88), *L. infantum* (78), *L. donovani* (78), *L. panamensis* (88)

^a^ The TriTrypDB ID of the protein containing the epitope has been provided in the Supplementary Table S1. ^b^ DTUs of *Trypanosoma cruzi* in which the epitope sequences were conserved 100%. ^c^ The percentage reported corresponds to the sequence identity of the nine residues among the protein sequences of kinetoplastid species.

**Table 2:** Molecular docking of the novel epitopes to HLA-A*02:01 molecule.

		Molecular Docking in CABSDock and FireDock		
ID	Epitope	RSMD (Å)	Interactions	Binding Affinity (KJ/mol)
Tc07	FLLHLSLNV	0.42	23	−88.68
Tc11	ILCDFLLHV	0.49	28	−133.28
Tc17	KLWAFLWSI	**3.00** ^a^	31	−82.02
Tc18	LLMDCAAYL	1.46	23	**−64.49** ^a^
TC19	LLMDDFSAV	2.81	30	−126.11
Tc21	MLLLALAYI	1.77	28	−95.53
Tc29	VMMPLIFLI	1.43	33	−110.7
Tc32	YLIPISLFV	2.64	**22** ^a^	−112.07
Tc34	YLLPLLHTV	2.85	24	−99.21

^a^ Cut-off value to define a strong binding of the epitopes with HLA-A*02:01 in the molecular docking analysis.

## Data Availability

The original contributions presented in this study are included in the article/Supplementary Materials. Further inquiries can be directed to the corresponding authors.

## Supplementary Materials

The following supporting information can be downloaded at: https://www.mdpi.com/article/10.3390/pathogens14040342/s1, Figure S1. Predicted *T. cruzi* epitopes for HLA-A*02:01 allele (A). Average predicted HLA-A*02:01 affinity peptides predicted in RankPep and NetMHC. (B) Distribution of predicted peptides based on the number of programs. Epitopes from gray bars were selected for subsequent analysis. Figure S2. Expression and purification of recombinant multi-epitope protein. (A) Expression analysis of multi-epitope protein (~14.4 kDa) in *E. coli* Rosetta™ 2(DE3) and BL21 Star™ (DE3) from whole cell lysate. M: Molecular eight marker; lane 1and 4: uninduced; lane 2 and 5: after 0.5 mM IPTG induced for 16 h at 15 °C; lane 3 and 6; for 4 h at 37 °C. (B) Purification analysis of multi-epitope protein as of the whole cell lysate coming from *E. coli* Rosetta™ 2(DE3), after 0.5 mM IPTG induced for 16 h at 15 °C. M: marker; lane 1: flow through; lane 2: elution with 50 mM Tris-HCl, 0.15 M NaCl, 1 mM TCEP, 8 M urea, 300 mM imidazole. (C) The multi-epitope protein was expressed in *E. coli* Rosetta™ and analyzed by western blot analysis of multi-epitope protein with anti-His-tag monoclonal antibody. M: marker; lane1: purified multi-epitope protein. Supplementary Table S1. Predicted *T. cruzi* epitopes for HLA-A*02:01.
