# Supplementary material for: Immunogenicity of Trypanosoma cruzi Multi-Epitope Recombinant Protein as an Antigen Candidate for Chagas Disease Vaccine in Humans
Source: Pathogens. 2025 Apr 3;14(4):342. doi: 10.3390/pathogens14040342 (PMC12030589; doi:10.3390/pathogens14040342)
Supplement: Supplementary file 1 [file pathogens-14-00342-s001.zip › pathogens-3522856-supplementary table.pdf]

Supplementary data

Supplementary Table S1. Predicted *T. cruzi* epitopes for HLA-A*02:01.

ID	Epitope	Start[a]	Rank[b]	Gene ID[c]	Function
Tc1	ALLQFLLAV	373	1.5 (13)	TcCLB.503973.31	Hypothetical protein
Tc2	ALNESLSSL	693	3.1 (12)	TcCLB.510901.220	Flagellum attachment zone protein 20
Tc3	ALWSGFTSI	14	2.6 (16)	TcCLB.510889.10	Hypothetical protein
Tc4	FILDAPVAV	13	2.8 (16)	TcCLB.510329.50	Hypothetical protein
Tc5	FLAAAPLFV	749	3.6 (12)	TcCLB.510901.220	Flagellum attachment zone protein 20
Tc6	FLAGYNATV	65	3.4 (12)	TcCLB.510303.80	Kinesin, putative
Tc7	FLLHLSLNV	314	2.8 (12)	TcCLB.508273.10	Adenosine monophosphate deaminase-like protein, putative
Tc8	FLSESLEQL	648	2.1 (12)	TcCLB.510329.50	Hypothetical protein
Tc9	GLMQGMFAL	106	2.1 (12)	TcCLB.509569.110	Carrier protein, putative
Tc10	ILASVFFGV	40	1.3 (12)	TcCLB.444777.10	Drug resistance protein, putative
Tc11	ILCDFLLHV	222	2.1 (12)	TcCLB.503657.20	Bardet-Biedl síndrome 8 protein
Tc12	ILLEAIYKL	139	1.7 (12)	TcCLB.503861.10	Retrotransposon hot spot (RHS) protein, putative
		135	2.9 (12)	TcCLB.509429.4	
Tc13	ILVSFYITV	249	3.5 (12)	TcCLB.510729.290	Sphingomyelin/ceramide phosphorylethanolamine synthase, bifunctional
Tc14	KLDGVLTQV	319	3.0 (12)	TcCLB.509429.4	RHS protein, putative
Tc15	KLFDFFWRI	354	2.8 (13)	TcCLB.508433.80	Hypothetical protein
Tc16	KLLQNMFEV	351	2.7 (12)	TcCLB.511367.170	Vacuolar protein sorting-associated protein 35, putative
Tc17	KLWAFLWSI	292	3.5 (12)	TcCLB.508799.270	Protein associated with differentiation 8, putative
		357	3.4 (12)	TcCLB.509713.20	
		292	3.8 (12)	TcCLB.509713.10	
Tc18	LLMDCAAYL	165	3.6 (12)	TcCLB.508427.10	Hypothetical protein
Tc19	LLMDDFSAV	112	4.7 (13)	TcCLB.511727.100	Alpha/beta hydrolase, putative
Tc20	LLMNVIFLV	151	1.6 (12)	TcCLB.507765.120	Serine incorporator, putative
Tc21	MLLLALAYI	110	2.1 (12)	TcCLB.507519.190	Protein kinase (pseudogene), putative
Tc22	MLMEAAVEL	300	1.9 (14)	TcCLB.511827.80	Hypothetical protein
Tc23	RLLEEIINL	30	3.7 (12)	TcCLB.511843.80	RHS protein, putative
Tc24	RLMMSYFEV	275	3.7 (12)	TcCLB.509431.10	Hypothetical protein
Tc25	SMIAPLWSV	444	3.4 (12)	TcCLB.511503.9	Hypothetical protein
Tc26	TLMSFSIFV	275	2.3 (12)	TcCLB.508267.10	Hypothetical protein
Tc27	VLFTTSAHV	132	4.0 (13)	TcCLB.508475.20	Hypothetical protein
Tc28	VLQELLYLV	662	1.8 (12)	TcCLB.510355.250	Hypothetical protein

Tc29	VMMPLIFLI	468	3.7 (12)	TcCLB.509713.10	Protein associated with differentiation 8, putative
Tc30	YLADVFVIL	224<br>426	2.9 (16)<br>3.8 (16)	TcCLB.508799.280<br>TcCLB.508801.10	Protein associated with differentiation 8, putative
Tc31	YLDDALDVV	39	2.3 (12)	TcCLB.508153.600	Actin-like protein, putative
Tc32	YLIPISLFV	349	3.9 (12)	TcCLB.503999.100	Phospholipid- transporting ATPase 1-like protein, putative
Tc33	YLLEVIVQV	285	1.7 (13)	TcCLB.511367.170	Vacuolar protein sorting-associated protein 35, putative
Tc34	YLLPLLHTV	1198	1.9 (12)	TcCLB.504131.140	Hypothetical protein
Tc35	YMLSRVAAV	920<br>65	4.0 (12)<br>1.9 (12)	M58466<br>TcCLB.506471.120	Trans-sialidase
Tc36	QLLEHLVEL	239	1.4 (12)	PWV20564.1	PFR-2, Paraflagellar rod protein 2
Tc37	FLYNRPLSV	677<br>640<br>743	2.7 (12)<br>3.9 (12)<br>5.0 (12)	tr|P9060<br>TcCLB.506471.120<br>M58466	ASP-2, Amastigote surface protein-2<br>Trans-sialidase
Tc38	VLLPSLFLL	817<br>920	3.5 (12)<br>4.0 (12)	TcCLB.506471.120<br>M58466	Trans-sialidase
Tc39	KLDEFTSRV	78	1.2 (12)	PWU92734.1	FCBa, flagellar calcium-binding 24 kDa protein
Tc40	TLLYATVEV	379	3.9 (12)	K49TcCLB. 504131.140	FL160, Flagellum-Associated Protein

[a] First amino acid position of the epitope in the protein.
[b] The average rank of prediction for the peptide calculated for each protein (number of programs that predicted the epitope).
[c] Gene ID of the protein containing the predicted epitope.
