# Supplementary material for: Immunogenicity of Trypanosoma cruzi Multi-Epitope Recombinant Protein as an Antigen Candidate for Chagas Disease Vaccine in Humans
Source: Pathogens. 2025 Apr 3;14(4):342. doi: 10.3390/pathogens14040342 (PMC12030589; doi:10.3390/pathogens14040342)

Supplementary Figure S1. **Predicted *T. cruzi* epitopes for HLA-A*02:01 allele** A). Average predicted HLA-A*02:01 affinity peptides predicted in RankPep and NetMHC. B) Distribution of predicted peptides based on the number of programs. Epitopes from gray bars were selected for subsequent analysis.

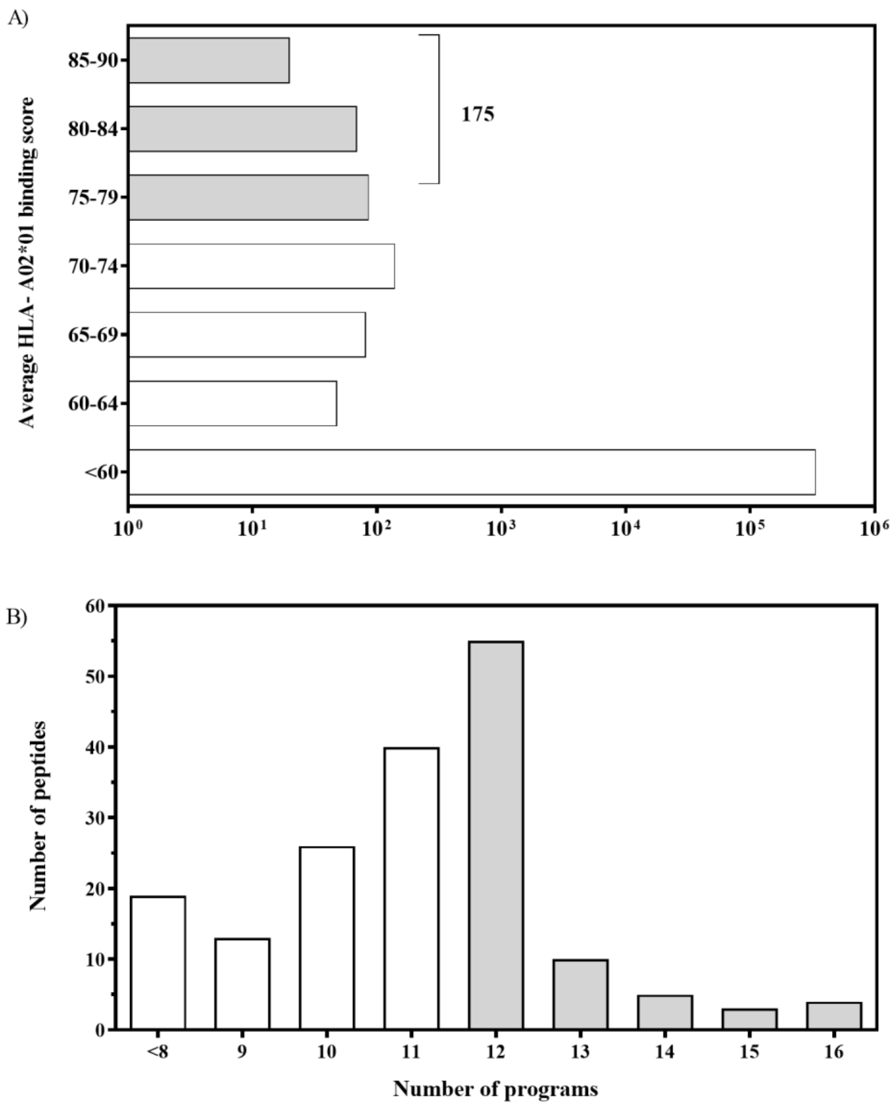

Supplementary Figure S2. Expression and purification of recombinant multi-epitope protein. A) Expression analysis of multi-epitope protein (~14.4 kDa) in *E. coli* Rosetta™ 2(DE3) and BL21 Star™ (DE3) from whole cell lysate. M: Molecular eight marker; lane 1and 4: uninduced; lane 2 and 5: after 0.5 mM IPTG induced for 16 h at 15°C; lane 3 and 6; for 4 h at 37°C. B) Purification analysis of multi-epitope protein as of the whole cell lysate coming from *E. coli* Rosetta™ 2(DE3), after 0.5 mM IPTG induced for 16 h at 15°C. M: marker; lane 1: flow through; lane 2: elution with 50 mM Tris-HCl, 0.15 M NaCl, 1 mM TCEP, 8 M urea, 300 mM imidazole. (C) The multi-epitope protein was expressed *in E. coli* Rosetta™ and analyzed by western blot analysis of multi-epitope protein with anti-His-tag monoclonal antibody. M: marker; lane1: purified multi-epitope

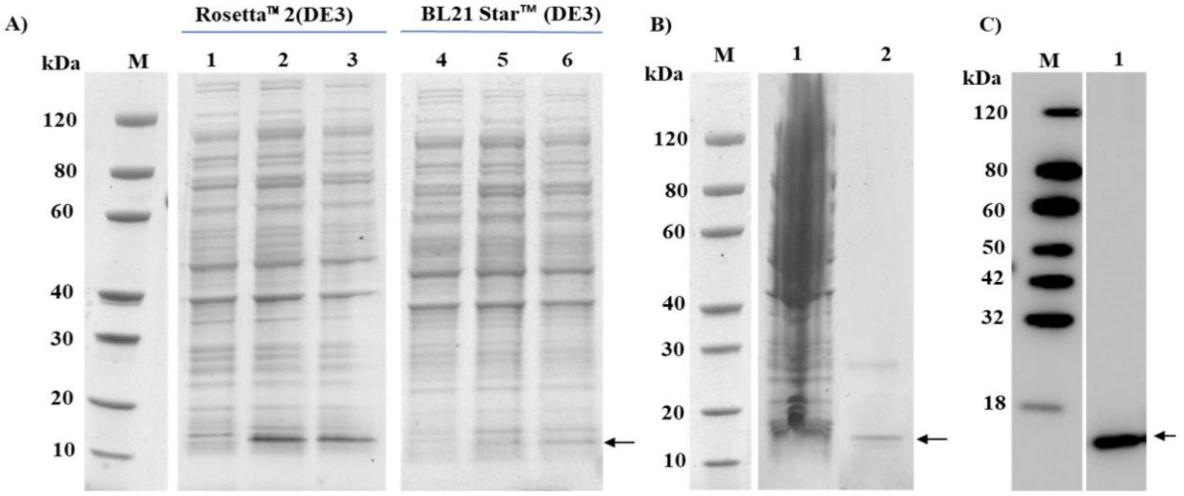

Supplement: Supplementary file 1 [file pathogens-14-00342-s001.zip › pathogens-3522856-supplementary.pdf]
